# Neural Innervation of Tumors: Mechanisms, Hallmarks, and Therapeutic Opportunities

**DOI:** 10.3390/cancers18071063

**Published:** 2026-03-25

**Authors:** Shamir Cassim, Christopher Montemagno

**Affiliations:** Département de Biologie Médicale, Centre Scientifique de Monaco, 98000 Monaco, Monaco; shamir_cassim@yahoo.fr

**Keywords:** tumor innervation, cancer neuroscience, neuron–tumor interactions, glioma, tumor microenvironment, neurotransmitter signaling, neural regulation of cancer, cancer progression

## Abstract

**Background/Objectives:** Increasing evidence indicates that tumors interact functionally with the nervous system. Rather than being passively innervated, many cancers establish bidirectional communication with neurons, suggesting that neural activity may represent an additional regulatory layer of tumor biology. This review aims to synthesize current knowledge on the mechanisms and consequences of tumor innervation and to discuss its implications for cancer progression and therapy. **Methods:** We performed a narrative synthesis of recent experimental and translational studies (2015–2026), identified through PubMed and major peer-reviewed biomedical journals. The literature was analyzed to identify key mechanisms of neural influence on tumor biology, including axonogenesis, pseudo-synaptic communication, neurotransmitter signaling, and metabolic coupling. **Results:** Emerging evidence indicates that neural inputs can regulate multiple hallmarks of cancer, including proliferation, invasion, angiogenesis, metabolic plasticity, and immune evasion. Tumors can actively recruit nerve fibers through axonogenic signals and establish specialized neuron–cancer interfaces that enable activity-dependent oncogenic signaling. In addition, neuronal interactions can influence tumor metabolism and therapeutic resistance through mechanisms such as mitochondrial transfer and neurotransmitter-driven signaling pathways. **Conclusions:** Tumor innervation represents an important and increasingly recognized dimension of cancer biology. Understanding how neural circuits interact with tumor cells and the surrounding microenvironment may reveal new biomarkers and therapeutic strategies aimed at disrupting tumor–neuron communication.

## Simple Summary

Tumor development does not occur in isolation but involves complex interactions with multiple components of the body, including the nervous system. Recent studies have shown that nerve fibers can infiltrate tumors and communicate directly with cancer cells. Through chemical signals and electrical activity, neurons can influence tumor growth, invasion, metabolism, and immune responses. In this review, we provide an integrated and updated synthesis of the emerging field of cancer neuroscience. Beyond summarizing current knowledge, we introduce a unifying conceptual framework in which tumors are viewed as aberrantly innervated organs capable of actively engaging and exploiting neural signaling. We highlight novel aspects of neuro–tumor interactions, including activity-dependent signaling, pseudo-synaptic communication, and intercellular organelle transfer. Importantly, we propose that neuronal activity may act as a cross-cutting regulator of multiple hallmarks of cancer, rather than affecting isolated processes. We also discuss how these insights open new therapeutic perspectives aimed at disrupting nerve–tumor communication. By emphasizing these emerging concepts and integrating them into a cohesive model, this review provides new insights into the neural dimension of cancer and its potential implications for future research and treatment strategies.

## 1. Introduction

The nervous system is emerging as a previously underappreciated regulator of cancer biology [1,2], following seminal work demonstrating that autonomic nerves actively promote tumor progression in prostate cancer [3]. More broadly, the role of nerves in tissue homeostasis has long been established, encompassing the regulation of development, regeneration, inflammation, and metabolic adaptation. In this context, it is perhaps not surprising that similar neurobiological principles may be co-opted by tumors to support their growth and evolution. However, this paradigm has only recently gained traction within oncology.

For decades, tumors were primarily conceptualized as genetically driven cell populations shaped by stromal, vascular, and immune interactions [4,5]. Although nerve fibers were frequently observed within tumors, they were largely considered incidental features of tissue remodeling rather than functional contributors to malignancy [2]. This view has now fundamentally shifted with the emergence of the cancer neuroscience field.

Accumulating evidence now demonstrates that tumors are not merely innervated structures but actively establish bidirectional communication with peripheral and central neurons [2,6]. Across multiple tumor types, mechanistic studies have identified functional neuron–cancer synapses (called pseudo-synapses in the following parts of the manuscript), activity-dependent oncogenic signaling, and intercellular organelle transfer from neurons to cancer cells [1,2]. These findings reveal that neuronal inputs can regulate proliferation, survival, invasion, metastasis, metabolic adaptation, immune modulation, and therapeutic resistance [7]. Rather than acting on isolated pathways, the nervous system appears to orchestrate multiple dimensions of tumor biology in a coordinated manner. This raises the possibility that neural signaling may function as a systems-level regulator of malignancy, integrating environmental cues with tumor-intrinsic signaling networks.

Despite rapid advances in cancer neuroscience, a unifying framework integrating tumor innervation, neuronal activity, and oncogenic signaling remains incomplete [6,7]. The field remains fragmented across tumor types and mechanistic domains, highlighting the need for conceptual consolidation and careful distinction between experimentally demonstrated mechanisms and emerging hypotheses [6,7].

In this review, we synthesize current knowledge to propose a cohesive model in which tumors function as aberrantly innervated organs. We examine the molecular architecture of tumor-directed axonogenesis, neuronal reprogramming, and pseudo-synaptic interface formation, and we analyze how neural activity converges on canonical oncogenic signaling networks. By mapping neuronal regulation onto the established hallmarks of cancer, we argue that neural control constitutes a cross-cutting and integrative dimension of malignancy with important therapeutic implications.

## 2. Foundations and Molecular Architecture of Tumor Innervation

### 2.1. From Passive Innervation to Active Neural Integration

Tumor-associated nerves were long regarded as passive bystanders, with heterogeneous innervation patterns attributed to inflammation or nonspecific tissue remodeling [6,7,8]. This view has been fundamentally revised by advances in high-resolution imaging, including intravital multiphoton microscopy, three-dimensional tissue clearing, and light-sheet imaging [7]. Together, these approaches demonstrate that tumors actively recruit, spatially organize, and functionally integrate neuronal elements within the tumor microenvironment [7].

Electrophysiological recordings further reveal that tumor-infiltrating neurons retain spontaneous and stimulus-evoked firing properties and that their activity influences tumor behavior in a frequency- and pattern-dependent manner [9,10]. These findings indicate that neuronal inputs are not merely structural features but rather constitute active signaling components capable of dynamically modulating malignant phenotypes [9,10].

Contemporary reviews and experimental studies demonstrate that cancers actively recruit peripheral nerve fibers into the tumor microenvironment through tumor-derived neurotrophic factors and axon guidance molecules, leading to increased intratumoral innervation rather than passive bystander presence [11,12,13]. These pseudo-synapse networks enable both rapid and spatially restricted communication [11,14]. Collectively, these observations support a conceptual shift in which tumors are best viewed as aberrantly innervated organs, wherein neurons actively participate in shaping the malignant niche by integrating electrical, chemical, and paracrine signals that regulate proliferation, survival, and metastatic competence.

### 2.2. Tumor-Induced Axonogenesis and Neurogenesis

Tumor-induced axonogenesis has been demonstrated in multiple experimental systems, showing that factors released by cancer cells can directly stimulate neurite outgrowth from neurons [7,15]. In a mouse model and in vitro studies, cancer cell–derived exosomes were shown to induce neurite extension from PC12 cells and sensory neurons; pharmacological blockade of exosome release in vivo significantly reduced nerve ingrowth into tumors, demonstrating that tumor-released vesicles and their cargo, including guidance molecules such as EphrinB1, can directly drive axonal recruitment toward the tumor mass [16]. Also, a recent work of Kobayashi et al. has identified Netrin-1 as a tumor-secreted axon guidance cue that actively promotes sympathetic axonogenesis through engagement of the NEO1 receptor. Genetic ablation of Ntn1 in tumor models markedly reduced intratumoral sympathetic innervation, whereas exogenous Netrin-1 enhanced axonal extension from celiac ganglia, establishing tumor-derived Netrin-1 as a direct molecular driver of cancer-induced axonogenesis [17].

Tumor cells and stromal cells within the tumor microenvironment have also been shown to secrete neurotrophic proteins that act on neurons [7,15]. Members of the neurotrophin family, including nerve growth factor (NGF), brain-derived neurotrophic factor (BDNF), and neurotrophin-3 (NT-3) are well characterized in neuronal biology for their roles in promoting neurite growth and neuronal survival [18,19]. Cultured dorsal root ganglion neurons exhibit significant neurite elongation in response to NGF and NT-3, with increased linear outgrowth quantified in primary neuronal cultures exposed to these factors, indicating that these neurotrophic ligands can directly modulate axonal extension in a dose-dependent manner [20]. Tumor stroma and cancer cells express neurotrophic factors and cytokines linked to neurite growth—for example, stromal cells derived from osteosarcoma secrete BDNF and IL-6 at levels that significantly promote axonal outgrowth from sensory neurons in culture, and neutralizing antibodies against BDNF or IL-6 markedly reduce this tumor-associated axonogenesis, definitively linking tumor microenvironment secretions to neural growth [21].

In addition to axonal recruitment, tumor-associated neurogenesis—defined as the emergence or expansion of neuronal elements within the tumor niche—has been directly observed using lineage tracing techniques. In a prostate cancer model, subventricular zone–derived neural progenitor cells were shown to migrate to the tumor site, differentiate into sympathetic and parasympathetic neurons, and contribute to functional nerve networks [22]. The depletion of these progenitor populations attenuated tumor growth and progression, whereas their transplantation enhanced tumor xenograft growth and metastatic spread, establishing a causal role for tumor-induced neurogenesis in vivo [22].

Taken together, these findings establish that tumors can actively construct and organize their own innervation through axonogenesis and neurogenesis, shaping a functional neural network within the malignant niche. Rather than implying deliberate architectural control, these processes likely reflect adaptive cellular interactions between tumor cells, stromal elements, and neural progenitors. Future studies should aim to map the precise spatiotemporal dynamics of tumor-induced neural remodeling and define the full spectrum of tumor-derived cues driving neural remodeling. Conceptually, these observations support the view that tumor–neural interactions can generate structured innervation patterns that may reinforce growth, plasticity, and therapy adaptation.

### 2.3. Neuronal Reprogramming by Tumor Cells

Neurons present within the tumor microenvironment have been shown to respond to tumor-derived signals, including cytokines, extracellular vesicles, and growth factors [23]. These signals can modulate neuronal phenotype, excitability, and transcriptional programs, suggesting that tumor-associated nerves undergo functional reprogramming rather than remaining passive structural elements. Tumor-derived factors such as nerve growth factor (NGF), brain-derived neurotrophic factor (BDNF), and inflammatory mediators have been implicated in promoting neuronal survival, axonal sprouting, and increased synaptic activity within the tumor microenvironment [23].

In experimental models of pancreatic ductal adenocarcinoma (PDAC), tumor cells have been shown to establish pseudo-synaptic glutamatergic connections with sensory neurons, supporting direct and functional neuron–cancer communication [24]. These structures display key features of synaptic organization, including vesicular glutamate release and receptor-mediated signal transduction, indicating that tumors can engage canonical neuronal communication mechanisms. Such interactions are not limited to structural connectivity but are functionally relevant, as neuronal activity can enhance tumor cell proliferation and invasive behavior. These findings indicate that tumor cells can actively remodel local neuronal circuits and promote sustained excitatory signaling within the tumor microenvironment [24].

Emerging evidence suggests that tumors may influence neuronal function through metabolic and bioenergetic coupling. Metabolic interactions within the tumor microenvironment have been documented extensively between cancer cells and stromal components, including nutrient exchange, mitochondrial remodeling, and adaptation to hypoxic conditions [25]. Although most studies have focused on tumor–stromal interactions, similar principles may extend to tumor–neuron crosstalk. Tumor-derived signals could potentially alter neuronal metabolic states, affecting mitochondrial dynamics, oxidative phosphorylation, and neurotransmitter synthesis, thereby modulating neuronal activity in a manner that supports tumor progression.

Collectively, these observations support a model in which neurons within the tumor microenvironment are not only structurally integrated but also functionally reprogrammed by tumor-derived cues. This bidirectional interaction contributes to the establishment of a dynamic neuro–tumor niche, where neuronal activity and tumor cell behavior are tightly interconnected.

### 2.4. Neurotransmitter Receptors and Ion Channels by Tumor Cells

Tumor cells have been shown to express a broad repertoire of neurotransmitter receptors and ion channels that are functionally active in multiple cancer types [26]. Ionotropic and metabotropic glutamate receptors (NMDA, AMPA, kainate, and mGluRs) are expressed in gliomas and several peripheral tumors, where their activation induces intracellular calcium influx and engages signaling pathways including MAPK/ERK and PI3K–AKT [27,28,29,30]. Calcium-dependent mediators such as CaMKII and calcineurin have also been reported downstream of glutamatergic signaling in cancer cells [29,30].

Cholinergic receptors, including muscarinic (M1–M5) and nicotinic subtypes, are similarly expressed in diverse malignancies [26,31]. Their activation has been shown to stimulate PLC–PKC signaling cascades and modulate intracellular calcium dynamics, influencing proliferation, migration, and survival in experimental models [31,32]. Adrenergic β1, β2, and β3 receptors are widely documented in solid tumors and couple to cAMP/PKA signaling and CREB-dependent transcriptional programs, with demonstrated effects on angiogenesis, invasion, and stress adaptation [33]. Purinergic P2X and P2Y receptors further contribute to calcium signaling, autocrine feedback loops, and inflammasome-associated inflammatory responses within the tumor microenvironment [34]. In addition, voltage-gated calcium, sodium, and potassium channels are expressed in various cancers, where they regulate membrane excitability, calcium homeostasis, and invasive behavior [35,36,37]. In specific contexts such as high-grade glioma, these structures have been described as providing direct evidence of functional electrochemical coupling [38]. In other tumor types, receptor and channel activation has been primarily characterized through pharmacological or genetic manipulation rather than through direct demonstration of physiological neuronal firing patterns driving oncogenic signaling in vivo [39]. Future studies will be required to determine whether, beyond receptor activation alone, tumor cells integrate endogenous neuronal activity in vivo in a temporally patterned manner, and whether such coupling feeds back to reshape local neural circuits.

### 2.5. Activity-Dependent Transcriptional Control

Experimental evidence indicates that neuronal activity can influence transcriptional programs in tumor cells through secreted factors [40]. In glioma models, neuronal activity promotes the release of neuroligin-3, which in turn activates PI3K–mTOR signaling and downstream gene expression programs that support tumor proliferation [40]. In ovarian carcinoma, adrenergic signaling triggered by norepinephrine has been shown to upregulate the EMT transcription factor Slug, enhancing cellular migration and invasion, while pharmacological inhibition of β2-adrenergic receptors reduces these effects [41]. Although these studies demonstrate that tumor cells can respond to neuronal or neurotransmitter-derived signals, direct evidence that neuronal firing patterns specifically induce activation of transcription factors such as CREB or NFAT, or that differential firing frequencies translate into distinct transcriptional outputs in vivo, remains limited.

### 2.6. Metabolic Coupling and Mitochondrial Transfer

Interactions between nerves and tumor cells have been shown to influence tumor cell metabolism through defined and experimentally documented mechanisms [25]. Several tumor types express functional neurotransmitter receptors, including glutamatergic (NMDA and AMPA), muscarinic cholinergic, purinergic, and adrenergic receptors [42]. Activation of these receptors can induce intracellular calcium transients or engage cAMP/PKA signaling pathways in cancer cells. These signaling cascades are known to regulate proliferation, survival, and mitochondrial function in various experimental models [43]. However, direct in vivo evidence demonstrating that neuronal electrical activity globally coordinates metabolic fluxes in tumor cells remains limited. Adrenergic β-adrenergic receptor signaling has been reported to influence mitochondrial dynamics and biogenesis through cAMP-dependent pathways, including regulation of PGC-1α–associated transcriptional programs [44]. In parallel, neural signaling can modulate stromal compartments within the tumor microenvironment. For example, sympathetic activation has been shown to stimulate lipolysis in adipocytes, increasing local fatty acid availability that may be utilized by tumor cells for energy production or membrane biosynthesis [45]. In addition, direct metabolic coupling between neurons and cancer cells has been demonstrated through mitochondrial transfer [25]. Preclinical studies have shown that neurons can donate mitochondria to adjacent tumor cells via contact-dependent mechanisms, resulting in enhanced oxidative phosphorylation (OXPHOS) capacity and increased bioenergetic fitness in recipient cancer cells (please refer to Section 4.5 for detailed discussion) [25]. These findings support the concept that mitochondrial function in tumors is dynamically regulated and can be influenced by neural components of the microenvironment.

Conceptually, one could hypothesize that tumors might progressively exploit surrounding neural networks to support their metabolic demands. Whether neural circuit dynamics are directly instrumentalized by tumors, however, remains to be formally demonstrated. This hypothesis requires experimental validation in future investigations to establish its causal relevance.

## 3. Neuron—Cancer Interfaces and Activity-Dependent Tumor Control

### 3.1. From Paracrine Signaling to Direct Cellular Interfaces

Initial models of neural influence in cancer emphasized paracrine communication, whereby neurotransmitters, neuropeptides, and neuromodulators diffuse across the tumor microenvironment to modulate cancer cell behavior. Although diffusion-based signaling remains relevant, converging evidence now supports the existence of specialized neuron–cancer junctions—commonly termed pseudo-synapses—that enable spatially confined and high-fidelity signal transmission [11,14]. High-resolution structural and functional studies have demonstrated that tumor-infiltrating neurons form synapse-like contacts (pseudo-synapses) with cancer cells, characterized by tight appositions between axonal terminals and tumor cell membranes, enriched in presynaptic and postsynaptic markers [46,47,48]. These interfaces permit rapid, spatially restricted signaling, enabling neuronal activity to influence tumor cell behavior in a manner analogous to canonical synapses, while exhibiting molecular plasticity and structural heterogeneity adapted to the tumor microenvironment [46,47,48]. Conceptually, this shift from diffuse neuromodulation to structured cellular interfaces reframes neuron–tumor communication as a direct and dynamically regulated signaling axis.

### 3.2. Molecular Composition of Neuron–Cancer Pseudo-Synapses

**Figure 1:**
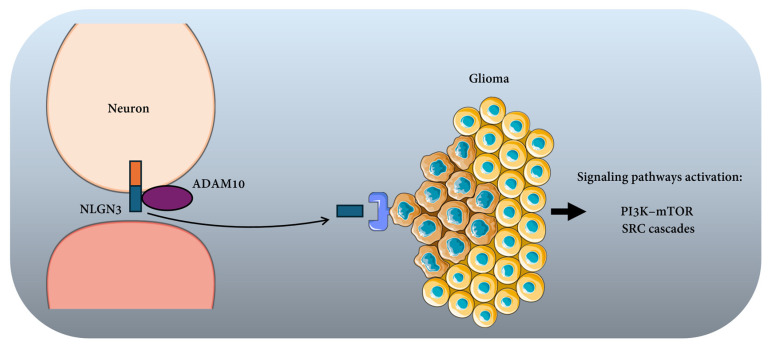
Neuron–glioma signaling mediated by Neuroligin-3 (NLGN3).

Recent research has identified specific molecular components of the interfaces formed between neurons and tumor cells in the brain, particularly in gliomas. Electrophysiological and ultrastructural studies have demonstrated functional synaptic contacts between excitatory neurons and glioma cells, in which canonical synaptic molecules are recruited to mediate communication [14,46]. A central molecule identified in this context is neuroligin-3 (NLGN3), a postsynaptic adhesion protein normally involved in trans-synaptic interactions with presynaptic neurexins (please refer to Section 3.4 for more details) [14]. Neuronal activity induces ADAM10-dependent cleavage and shedding of NLGN3, releasing its ectodomain into the tumor microenvironment, where it activates oncogenic signaling pathways in glioma cells, including PI3K–mTOR and SRC cascades [14] (Figure 1). These findings establish that at least part of the classical synaptic adhesion apparatus contributes to neuron–tumor communication. Although additional synaptic adhesion families have not yet been definitively characterized at these interfaces in vivo, current evidence indicates that neuron–cancer pseudo-synapses co-opt canonical synaptic molecules to enable direct and activity-dependent signaling within the tumor microenvironment [46].

Schematic representation of the mechanism by which neuronal activity promotes glioma growth through the release of Neuroligin-3 (NLGN3). In neurons, the membrane-bound NLGN3 protein is proteolytically cleaved by the metalloprotease ADAM10, resulting in the release of soluble NLGN3 into the extracellular space. The secreted NLGN3 acts on neighboring glioma cells and activates oncogenic intracellular signaling pathways, including the PI3K–mTOR signaling pathway and SRC kinase signaling cascade. Activation of these pathways enhances tumor cell proliferation and contributes to glioma progression. This model highlights the role of neuron-tumor interactions in the regulation of glioma growth and identifies NLGN3-dependent signaling as a potential therapeutic target.

### 3.3. Neurotransmitter-Specific Signaling Modalities

Distinct neurotransmitter systems contribute to tumor progression in a context-dependent yet mechanistically convergent manner. Glutamatergic signaling acts as a dominant driver in multiple malignancies, including PDAC. Activation of NMDA and AMPA receptors induces calcium influx, engaging MAPK/ERK, PI3K–AKT, CaMKII, and calcineurin pathways that promote proliferation, invasion, and stress resistance. Metabotropic glutamate receptors further reinforce metabolic adaptation and apoptotic resistance [24,27,49,50].

Cholinergic signaling through muscarinic and nicotinic receptors modulates intracellular calcium and PLC–PKC pathways, influencing proliferation and therapeutic resistance [32,43,51]. Adrenergic β-receptor activation engages cAMP/PKA–CREB signaling, promoting angiogenesis, invasion, and metabolic adaptation [33,52,53]. Purinergic P2X/P2Y receptors further couple calcium flux to inflammatory remodeling within the tumor microenvironment (for more details, please refer to Section 2.4) [54,55,56,57].

Despite their apparent diversity, these neurotransmitter systems converge on shared calcium-dependent hubs, rendering intracellular calcium the central integrator of neural influence. As a finely tuned second messenger, calcium decodes the intensity and rhythm of neuronal signals and converts them into coordinated proliferative, metabolic, and survival responses—functioning as a central regulator of the neural–tumor axis.

### 3.4. Neuronal Activity as an Oncogenic Signal

Neuronal firing itself constitutes a potent oncogenic stimulus in high-grade glioma, illustrating that electrical activity can directly regulate tumor growth. In the seminal study by Venkatesh et al. [40], neuronal activity was experimentally manipulated in orthotopic xenograft mouse models of human glioma. Optogenetic stimulation of cortical neurons significantly increased tumor cell proliferation in vivo, whereas pharmacological suppression of neuronal activity reduced tumor growth, establishing a causal relationship between neuronal activity and tumor progression [40].

Using acute cortical slice cultures, the authors further demonstrated that neuronal depolarization—induced either by optogenetic stimulation or elevated extracellular potassium—triggered the release of a soluble mitogenic factor. Biochemical analyses identified neuroligin-3 (NLGN3), a synaptic adhesion molecule, as this activity-dependent factor. Recombinant NLGN3 was sufficient to stimulate proliferation of patient-derived glioma cells and rapidly activated PI3K–mTOR signaling pathways. In addition, transcriptomic analyses revealed broad oncogenic reprogramming following NLGN3 exposure, including upregulation of genes involved in cell cycle progression, synaptic signaling, and tumor–microenvironment interactions [40]. Importantly, glioma growth was markedly impaired in Nlgn3 knockout mice, demonstrating that microenvironmental NLGN3 is not merely sufficient but required for tumor progression in vivo. This finding highlights the critical role of neuron-derived signals in sustaining glioma growth and supports the concept of activity-dependent trophic support within the tumor microenvironment [40].

The follow-up study of Venkatesh et al. defined the mechanism of activity-dependent NLGN3 release and tested therapeutic targeting. The authors demonstrated that neuronal activity induces proteolytic cleavage and shedding of NLGN3, primarily mediated by ADAM10 (Figure 1). Pharmacological inhibition of ADAM10 reduced NLGN3 secretion from active brain slices and significantly suppressed glioma growth in multiple patient-derived orthotopic xenograft models, phenocopying the growth defect observed in Nlgn3-deficient hosts. Notably, diverse molecular subtypes of high-grade glioma displayed dependency on microenvironmental NLGN3 [58].

Together, these studies demonstrate that neuronal electrical activity drives glioma progression through activity-dependent proteolytic release of NLGN3, which activates oncogenic signaling pathways to sustain tumor growth in vivo (Figure 1).

### 3.5. Neural Regulation of Invasion and Metastasis

Neuronal signaling contributes directly to tumor cell invasion and metastatic progression through calcium-dependent mechanisms activated downstream of glutamatergic and cholinergic inputs. Glutamatergic neuron-to-tumor communication has been shown to enhance invasive behavior via NMDA receptor (NMDAR) signaling. Zeng et al. have shown that brain metastatic cancer cells were found to localize in close synaptic proximity to glutamatergic neurons and exploit NMDAR-mediated calcium influx. Functional analyses demonstrated that NMDAR activation promoted metastatic outgrowth in the brain. Of note, genetic or pharmacological inhibition of NMDAR signaling impaired metastatic colonization [59]. Consistently, in vitro activation of NMDAR led to an increased activity of matrix metalloproteinase-2 (MMP-2), a key mediator of extracellular matrix degradation and invasion. NMDAR stimulation enhanced both proliferative and invasive properties, whereas receptor blockade using MK-801 reduced MMP-2 activity, directly linking glutamate-driven calcium entry to proteolytic remodeling mechanisms required during invasion [60].

Cholinergic signaling similarly regulates invasive behavior. Recently, Hering et al. have shown that activation of muscarinic M3 receptors promoted colorectal cancer progression, including enhanced migration and invasion. Pharmacological blockade of M3 signaling reduced invasive capacity in vitro and suppressed tumor progression in vivo, with associated modulation of pro-invasive signaling pathways and matrix-remodeling enzymes [61]. Together, these studies demonstrate that neuronal inputs—particularly glutamatergic and cholinergic signaling—activate calcium-dependent pathways that enhance matrix degradation, motility, and metastatic colonization, thereby positioning neural signaling as an active regulator of the metastatic cascade.

### 3.6. Integration with Stromal and Immune Components

Neuron–tumor communication extends beyond direct cancer cell interactions to encompass broad remodeling of the tumor ecosystem. Neural inputs regulate fibroblast activation, endothelial behavior, Schwann cell dynamics, and immune cell polarization, reshaping stromal architecture and inflammatory tone. Recent evidence further demonstrates that tumors engage in long-range, bidirectional communication with the central nervous system, establishing functional sensory–sympathetic circuits that actively promote tumor progression [6]. In this framework, tumor-derived signals recruit and activate sensory afferents—such as vagal pathways—which relay information to the brainstem [6]. Central integration of these signals enhances sympathetic outflow back to the tumor microenvironment, where adrenergic signaling suppresses antitumor immunity.

Sympathetic and sensory signaling orchestrate macrophage polarization toward tumor-supportive phenotypes, modulate dendritic cell maturation, and shape T cell trafficking. For example, in preclinical PDAC models, nociceptor neurons release the neuropeptide calcitonin gene-related peptide (CGRP), which activates RAMP1 receptors on cancer-associated fibroblasts, suppressing IL-15 and diminishing natural killer cell recruitment and cytotoxicity, thereby fostering an immunosuppressive niche that facilitates tumor progression [62]. Beyond CGRP, other neuropeptides—including substance P and neuropeptide Y—further reinforce this immune-permissive landscape [63,64]. Through these multidimensional interactions the nervous system emerges as a key integrative component of the tumor ecosystem, dynamically integrating electrical signaling and metastatic competence, thus positioning neural circuitry as an important player in malignant progression.

## 4. Neural Regulation Across the Hallmarks of Cancer

### 4.1. Sustaining Proliferative Signaling

Neural inputs provide potent proliferative drive across diverse tumor contexts by directly coupling neuronal activity to core cell-cycle regulatory machinery. In colorectal cancer models, activation of muscarinic acetylcholine receptors—particularly the M3 subtype—engages intracellular proliferative pathways. Indeed, in vivo blockade of M3R with darifenacin significantly suppresses tumor cell proliferation, attenuates ERK1/2 and AKT signaling, and reduces primary tumor growth, establishing a mechanistic link between cholinergic signaling and oncogenic proliferation [61]. Also, in murine intestinal tumorigenesis, Peng et al. demonstrated that chronic stimulation of muscarinic receptors increases both tumor number and volume alongside upregulation of proliferative markers such as cyclin D1, underscoring the pro-mitogenic influence of cholinergic inputs on cell-cycle progression [65].

In parallel, glutamatergic signaling contributes to proliferative regulation in cancer cells through calcium-dependent mechanisms. In tumor cells expressing Ca^2+^-permeable AMPA-type glutamate receptors, activation of these receptors triggers rapid calcium influx that stimulates PI3K/AKT signaling, linking excitatory neurotransmission to enhanced cell survival and proliferation [66].

**Figure 2:**
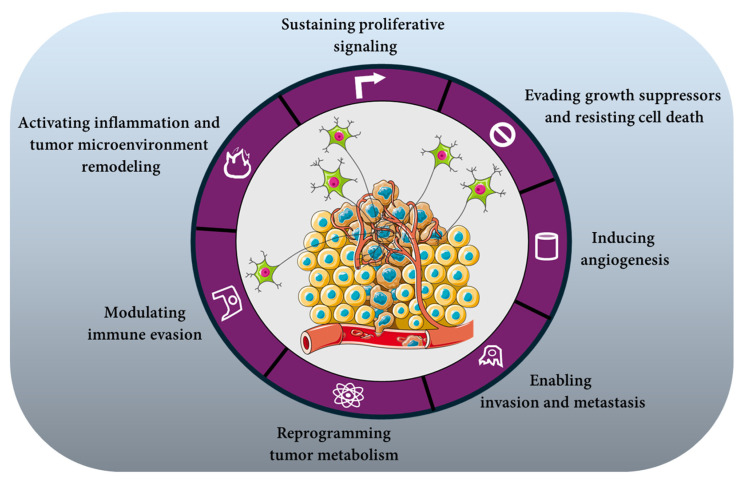
Neural–stromal interactions driving tumor progression in the tumor microenvironment.

Through these convergent calcium- and second messenger-dependent routes, neuronal regulation sustains proliferative signaling even amid microenvironmental adversity—nutrient limitation, hypoxia, or therapeutic stress (Figure 2). These observations support the view that neural inputs participate in sustaining proliferative capacity under adverse microenvironmental conditions.

### 4.2. Evading Growth Suppressors and Resisting Cell Death

Neural signaling can modulate apoptotic pathways in target cells by attenuating growth-suppressive signals and enhancing pro-survival programs. For example, in vivo studies in rats have shown that brain-derived neurotrophic factor (BDNF) dose-dependently increases Bcl-xL mRNA and protein levels via activation of CK2 and NF-κB in hippocampal neurons [67]. However, while these findings support a neural regulation of anti-apoptotic programs, direct evidence in tumor cells is still limited. In cervical cancer cells, activation of β_2_-adrenergic receptors (β_2_-AR) has been shown to promote cell survival through a defined signaling pathway. β-AR stimulation upregulates Sirt1, a deacetylase that reduces the acetylation and transcriptional activity of p53, thereby impairing the expression of pro-apoptotic genes and diminishing checkpoint-mediated apoptosis. This mechanism highlights how adrenergic signaling can weaken p53-dependent growth control and enhance tumor cell resistance to apoptotic stimuli [68]. Cholinergic and glutamatergic signaling have been shown to support tumor cell survival by engaging calcium-dependent pathways that converge on key pro-survival kinases. In glioma cells, activation of metabotropic glutamate receptors (mGluR1) was shown to stimulate the PI3K/Akt pathway, promoting cell viability [69]. Similarly, in lung cancer cells, nicotinic acetylcholine receptor (nAChR) activation by nicotine induced Akt and NF-κB signaling, increased cyclin D1 expression, and protected cells from apoptotic stimuli [70,71]. Muscarinic acetylcholine receptor (mAChR) stimulation in breast cancer cells also activated MAPK/ERK, supporting proliferation and survival [32]. These studies demonstrate that cholinergic and glutamatergic inputs can enhance tumor cell survival through coordinated activation of PI3K/Akt and MAPK/ERK pathways, although direct links to ROS-mediated apoptosis or cyclin stabilization in a single tumor model have not yet been established. Taken together, these findings highlight that diverse neural inputs—adrenergic, cholinergic, and glutamatergic—can converge on multiple pro-survival signaling networks in tumor cells, collectively enabling them to evade growth suppressors and resist cell death (Figure 2).

### 4.3. Inducing Angiogenesis

Neural inputs act as active regulators of tumor angiogenesis by aligning vascular growth with metabolic and hypoxic demands [72,73]. In preclinical models of pancreatic cancer cells, stimulation of β-adrenergic receptors with β-adrenergic agonists such as isoproterenol increases the accumulation of hypoxia-inducible factor-1α (HIF-1α) under normoxic conditions. This effect involves β-adrenergic receptor–dependent activation of downstream signaling pathways, including PKA, ERK1/2 and PI3K/Akt. The resulting stabilization and activation of HIF-1α enhances the transcription of HIF-1 target genes, including pro-angiogenic factors such as vascular endothelial growth factor (VEGF) [74]. Cholinergic signaling contributes to tumor-associated angiogenic responses in specific experimental models. In human MCF-7 breast cancer cells, activation of muscarinic acetylcholine receptors increases nitric oxide (NO) production through stimulation of nitric oxide synthase (NOS) activity. This is accompanied by enhanced VEGF expression and promotion of angiogenesis in vivo, as demonstrated in tumor xenograft models derived from MCF-7 cells. These findings support a mechanistic link between muscarinic receptor activation, NO signaling, and induction of pro-angiogenic pathways in this breast cancer model [75]. In addition, activation of the α7 nicotinic acetylcholine receptor on endothelial cells has been shown to promote endothelial cell proliferation, migration, and tube formation—key processes required for angiogenesis [76]. Together, adrenergic and cholinergic influences create a neurally gated vascular response in which the nervous system calibrates vessel formation to the evolving energetic and oxygen needs of the tumor (Figure 2).

Glutamatergic signaling via metabotropic glutamate receptor 1 (mGluR1) promotes angiogenic responses in experimental models. In particular, activation of mGluR1 in endothelial and cancer cells enhances formation of capillary-like structures in vitro and increases angiogenesis in tumor xenograft models, indicating that glutamate receptor–dependent signaling contributes to the induction of pro-angiogenic pathways [77]. In other cellular systems, glutamate receptor activation can induce calcium-dependent activation of transcription factors such as CREB, which regulate gene expression, though this mechanism has been demonstrated primarily in neuronal models rather than directly in cancer-associated angiogenesis [78].

Sensory neuropeptides also have a significant role in modulating angiogenic processes. In endothelial cells, substance P enhances NO production and promotes cell migration, supporting angiogenic responses in both in vitro and in vivo conditions [79]. Similarly, calcitonin gene-related peptide (CGRP) stimulates endothelial proliferation, further contributing to the expansion of the vascular network [80]. Moreover, in co-culture systems of sensory neurons and endothelial cells, neuronal signals upregulate the expression and activity of MMP2 and MMP9, thereby facilitating extracellular matrix remodeling and vascular sprouting [81]. Importantly, neural regulation extends beyond the primary tumor: innervation of distant tissues can prime pre-metastatic niches by modulating endothelial responsiveness and chemokine gradients that favor subsequent colonization. In this way, neural inputs coordinate both local vessel formation and systemic vascular adaptation, supporting the concept that neural inputs can contribute to both angiogenesis and systemic vascular priming in specific contexts (Figure 2).

### 4.4. Enabling Invasion and Metastasis

Neural inputs orchestrate metastatic progression by integrating cytoskeletal dynamics, adhesion, metabolic adaptation, and tissue tropism. A clear mechanistic link between neuronal activity and metastasis has recently emerged from studies showing that direct glutamatergic synaptic input from neurons to metastatic cells in the brain promotes early metastatic seeding and growth [59,82]. Indeed, in rodent models of breast cancer and melanoma brain metastasis, Zeng et al. showed that breast cancer cells exploit NMDAR-dependent signaling at neuron-tumor contacts (pseudo-synapse), enhancing survival and proliferation in the brain—Mondal et al. demonstrated more recently that blocking glutamatergic synaptic activity reduces metastatic burden, indicating that neuronal input actively drives metastatic progression [59,82].

Cell migration and invasion depend on dynamic regulation of cytoskeletal architecture and adhesion complexes, processes governed by small GTPases such as RhoA, Rac1, and Cdc42, which modulate lamellipodia, filopodia, and invadopodia formation during metastasis [83,84]. Although these pathways are well documented as critical effectors of migratory behavior, direct preclinical evidence linking specific neural signaling to Rho-GTPase–mediated invasion outside of the central nervous system remains an active area of investigation.

Beyond local invasion, gradients of neurotrophic factors such as nerve growth factor (NGF), brain-derived neurotrophic factor (BDNF), and glial cell-derived neurotrophic factor (GDNF) have been shown to establish chemotactic cues that attract tumor cells and support nerve-directed migration in peripheral tissues, creating permissive microenvironments for dissemination [85,86]. These neurotrophin gradients may function as chemotactic cues that bias circulating tumor cells toward receptive niches, illustrating how the nervous system can shape metastatic tropism.

Metabolic support provided by neural inputs—such as calcium-dependent modulation of cellular energetics—can further sustain the bioenergetic demands of motile tumor cells, while sympathetic signaling enhances vascular permeability through VEGF induction and remodeling of endothelial junctions, facilitating tumor cell intravasation into the circulation [25,87,88,89,90,91]. Together, these neural mechanisms weave a cohesive regulatory network in which neural signals influence motility, metabolic resilience, and spatial guidance, coordinating each stage of the metastatic cascade through precise cellular and molecular control (Figure 2).

### 4.5. Reprogramming Tumor Metabolism

Neural activity couples electrical and metabolic interactions at the nerve–tumor interface to support tumor bioenergetics and adaptability. A key preclinical study by Hoover et al. demonstrated that cancer-associated neurons enhance tumor metabolic plasticity by directly transferring mitochondria to adjacent cancer cells [25]. In in vitro coculture of aggressive 4T1 breast carcinoma cells with neurons bearing genetically labeled mitochondria, confocal microscopy and flow cytometry confirmed transfer of eGFP-labeled neuronal mitochondria into cancer cells via tunneling nanotube-like structures. The presence of a GFP+/mCherry+ double-positive cancer cell population indicated intercellular mitochondrial transfer, and inhibition of tunnelling nanotube formation reduced this transfer. To test the functionality of transferred mitochondria, ρ0 (mitochondrial DNA-deficient) 4T1 cells were cocultured with mitochondria-competent neurons; only cocultured ρ0 cells reacquired mitochondrial DNA, restored OXPHOS capacity, and formed viable colonies in the absence of uridine, demonstrating that transferred mitochondria were functional and capable of rescuing mitochondrial respiration and proliferative capacity. Lineage tracing with the MitoTRACER reporter system revealed that cancer cells that acquired neuronal mitochondria in primary tumors were selectively enriched at metastatic sites in vivo, indicating that mitochondrial transfer confers a metabolic advantage during metastatic dissemination.

**Figure 3:**
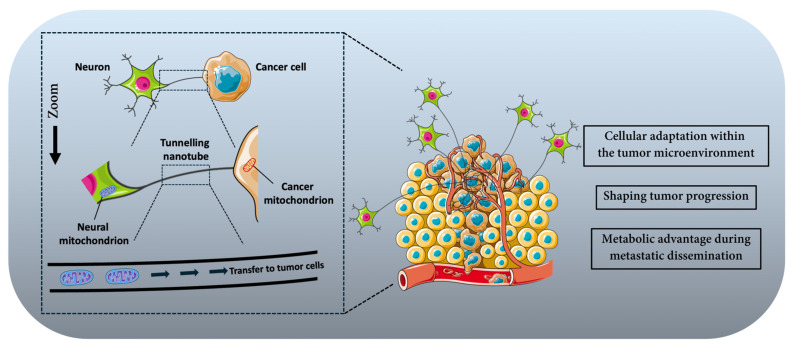
Neuron–tumor mitochondrial transfer through tunneling nanotubes [25].

These findings provide concrete evidence that neurons can act as metabolic donors to tumor cells, bolstering OXPHOS, ATP production, and resilience against metabolic stressors (Figure 3). By supplying functional organelles, neurons contribute to the metabolic adaptability of cancer cells, enabling them to maintain energy production and redox balance under microenvironmental stress and improving their capacity to survive and proliferate during metastatic progression.

Schematic representation of mitochondrial transfer from neurons to cancer cells within the tumor microenvironment. Neuronal cells can establish direct cytoplasmic connections with tumor cells through tunneling nanotubes [25]. Through these structures, neuronal mitochondria can be transported toward cancer cells, resulting in the transfer of functional mitochondria into tumor cells. This process of mitochondrial transfer contributes to metabolic reprogramming, enhanced bioenergetic capacity, and improved survival of cancer cells within the tumor microenvironment.

### 4.6. Modulating Immune Evasion

Neural regulation actively shapes tumor immune landscapes and can promote immune escape through multiple experimentally documented mechanisms. Activation of β-adrenergic signaling alters anti-tumor immunity by modulating the function and recruitment of immune cells within the tumor microenvironment. In murine melanoma models, blockade of β-adrenergic receptors with propranolol in combination with a peptide cancer vaccine enhanced peripheral dendritic cell maturation, increased the intratumoral proportion of effector CD8^+^ T cells, and decreased the presence of PD-L1^+^ myeloid-derived suppressor cells (MDSCs), demonstrating that sympathetic neurotransmission influences antigen-presenting cell function, T-cell infiltration, and immunosuppressive myeloid populations [92].

Independent work has shown that adrenergic stress enhances infiltration and differentiation of tumor-associated macrophages toward immunosuppressive phenotypes in breast cancer models, accompanied by elevated levels of pro-tumorigenic cytokines, consistent with sympathetic signaling steering macrophage polarization toward tumor-supportive states [93,94]. Importantly, emerging evidence indicates that tumor-associated macrophages (TAMs) do not merely function as immunosuppressive effectors but also actively contribute to tumor innervation [95]. Dolci et al. demonstrated that TAMs could promote neurite outgrowth through secretion of pro-neurogenic factors such as SPP1, engaging neuronal mTORC2 signaling pathways and facilitating axonal extension within the tumor microenvironment [95]. This establishes TAMs as direct architectural regulators of tumor-associated nerve infiltration. Additionally, chronic sympathetic innervation has been linked to reduced effector T-cell receptor signaling and diminished CD8^+^ T-cell responses, and adrenergic engagement can dampen macrophage-mediated inflammation while promoting phenotypes consistent with immune tolerance [96,97,98]. Sensory neuron-derived neuropeptides, such as calcitonin gene-related peptide (CGRP), have been implicated in modulating immune cell activity, with evidence that CGRP signaling in the tumor microenvironment influences macrophage cytokine production and reduces effector CD8^+^ T-cell activity [99,100,101]. Tumor-infiltrating nerves themselves can express immune checkpoint molecules such as PD-L1 in prostate cancer, correlating with lower CD8^+^ T-cell infiltration and adverse outcomes, indicating direct neural contributions to adaptive immune inhibition within tumors [102]. Neural modulation also affects regulatory T cells (Tregs): sympathetic nerve–released norepinephrine acting through β2-adrenergic receptors enhances the migration of Tregs into the tumor microenvironment, supporting their immunosuppressive functions [97,103,104].

Collectively, these findings position neural signaling as a central architect of the tumor immune microenvironment (Figure 2). Extending this framework, it could be hypothesized that tumor-associated innervation may also intersect with glyco-immune checkpoint signaling, contributing to a coordinated immunosuppressive axis. β-adrenergic signaling might influence tumor cell transcriptional programs via cAMP/PKA/CREB-dependent pathways, potentially regulating glycosyltransferases implicated in tumor hypersialylation [33]. Enhanced surface sialylation could increase engagement of inhibitory Siglec receptors on myeloid and lymphoid cells, thus reinforcing immune suppression [105,106,107]. Given that β-adrenergic and Siglec-mediated pathways may converge on shared downstream effectors—such as STAT3 activation, suppression of NF-κB–driven inflammatory responses, and induction of IL-10—a feed-forward neuro–glyco–immune circuit could plausibly emerge. In this context, the recent development of antibody–lectin chimeras targeting glyco-immune checkpoints by Stark et al. underscores the therapeutic relevance of glycan-mediated immunoregulation [108]. Should neural signaling prove capable of modulating tumor hypersialylation, combined targeting of neural pathways and glyco-immune checkpoints may represent a rational—albeit still speculative—strategy to disrupt this integrated immunosuppressive circuitry.

### 4.7. Activating Inflammation and Tumor Microenvironment Remodeling

Neural signaling remodels the tumor ecosystem by coordinating inflammatory and stromal programs through interactions with non-neoplastic cells in the tumor microenvironment [109]. Although the direct contribution of sympathetic nerves to specific cytokine induction within tumors remains under active investigation, emerging work shows that tumor stroma can secrete pro-inflammatory factors such as IL-6 that are associated with nerve recruitment and axonal outgrowth in osteosarcoma, indicating a functional link between inflammation, stromal signals, and neural elements in the tumor niche [21]. In this context, stromal production of IL-6 and neurotrophic factors supports both pro-inflammatory and neuritogenic processes that shape tumor progression [21].

Glial cells of the peripheral nervous system, particularly Schwann cells, are abundant components of the neural tumor microenvironment and have been shown to influence stromal composition and immune interactions [110,111,112]. Schwann cells within the tumor microenvironment secrete cytokines, chemokines, and other effector molecules that modulate the phenotype and function of innate and adaptive immune cells as well as fibroblasts, contributing to the establishment of an immunosuppressive milieu that facilitates tumor progression [110,111,112]. These neural–stromal interactions extend beyond soluble factors. Schwann cells migrate toward cancer cells even before overt tumor cell invasion of nerves and contribute to extracellular matrix reorganization and axon guidance within the tumor microenvironment through secretion of neurotrophic factors, chemokines, and matrix-associated proteins [112,113].

Together, these neural–stromal interactions integrate cytokine signaling, immune modulation, and extracellular matrix remodeling within the tumor microenvironment, thus contributing to the establishment of a dynamic and permissive niche (Figure 2).

Neural signaling contributes to the remodeling of the tumor microenvironment (TME) by coordinating inflammatory and stromal programs. These neural–stromal interactions contribute to multiple cancer-associated processes, including sustained proliferative signaling, evasion of growth suppressors and resistance to cell death, induction of angiogenesis, metabolic reprogramming, invasion and metastasis, immune modulation, and inflammatory remodeling of the TME. Together, these mechanisms highlight the functional interplay between neural elements, stromal cells, and inflammatory pathways in shaping tumor progression.

To provide a synthetic overview of these mechanisms, we summarize the main neural regulatory inputs across the hallmarks of cancer in Table 1.

## 5. Therapeutic Outlook and Combined Strategies

### 5.1. Targeting Neural Inputs in Cancer Therapy

Disrupting neuron–tumor communication constitutes a translational strategy supported by preclinical evidence [114]. Approaches include structural destabilization of neuron–tumor contacts (please refer to Section 3.2 for more details), pharmacological blockade of neurotransmission, suppression of neuronal activity, and interference with metabolic coupling [115,116]. Receptor-level inhibition has shown antitumor effects in experimental models. NMDA receptor antagonists reduce calcium-dependent MAPK/ERK activation and proliferation [60,117]. AMPA and mGluR inhibition impairs glutamate-driven growth. Muscarinic antagonists decrease proliferation and stemness-associated features, while β-adrenergic blockers (e.g., propranolol) attenuate cAMP/PKA–CREB signaling, angiogenesis, and metastasis [52,118,119,120,121]. Retrospective clinical studies report associations between β-blocker use and improved outcomes in selected breast and pancreatic cancer cohorts [122,123,124]. Metabolic interactions can also be targeted: inhibition of mitochondrial transfer reduces OXPHOS and increases chemotherapy sensitivity in preclinical models, and blockade of adrenergic signaling limits stromal lipolysis and nutrient availability [25]. Collectively, these findings support the rationale for targeting neural signaling as a component of tumor biology.

### 5.2. Modulating Pseudo-Synaptic Interfaces

In glioblastoma, functional glutamatergic synapses between neurons and tumor cells have been directly demonstrated using electrophysiology, electron microscopy, and optogenetic stimulation [125]. These neuron–glioma pseudo-synapses involve AMPA receptor–mediated currents in tumor cells and depend on postsynaptic scaffolding proteins required for receptor clustering. Neuronal activity increases tumor cell depolarization and promotes proliferation in vivo, establishing a causal link between synaptic transmission and tumor growth [48,125].

Neuroligin-3 (NLGN3), a synaptic adhesion molecule released in an activity-dependent manner by neurons and oligodendrocyte precursor cells, has been shown to stimulate glioma proliferation. Genetic deletion or pharmacological inhibition of NLGN3 shedding significantly reduces tumor growth in preclinical models, demonstrating that specific synaptic components can function as tumor growth regulators [40,58].

Future studies could determine whether additional synaptic adhesion molecules, such as neurexins or LRRTMs, play functional roles in pseudo-synaptic interfaces beyond NLGN3. It would also be important to assess whether these pseudo-synaptic assemblies exhibit molecular features sufficiently distinct from canonical neuronal synapses to allow selective therapeutic targeting. If such structural vulnerabilities were identified, strategies aimed at destabilizing receptor clustering or synaptic adhesion might modulate neuron-driven tumor support while preserving physiological neurotransmission.

### 5.3. Neuromodulatory Interventions

Experimental modulation of neuronal activity has demonstrated functional effects on tumor progression in preclinical models [126,127,128]. Saloman et al. reveals that ablation of sensory neurons in PDAC reduces primary tumor growth and progression to invasive carcinoma, as well as metastatic dissemination [128]. This intervention supports a causal contribution of neuronal excitability to tumor progression. In preclinical breast cancer models, pharmacological sympathectomy reduces intratumoral norepinephrine levels, indicating that sympathetic fibers are a major source of adrenergic signaling within the tumor microenvironment [129,130]. Experimental and stress-based models further demonstrate that β-adrenergic signaling promotes tumor growth, angiogenesis, and metastatic progression, thus pinpointing a functional role for sympathetic innervation in regulating breast tumor progression [129,130]. Electrical or genetic modulation of neural inputs also influences immune parameters within the tumor microenvironment. In murine melanoma and other cancer models, adrenergic signaling impairs antitumor immunity, particularly by suppressing CD8^+^ T-cell metabolic activation and effector functions. Pharmacologic attenuation of β-adrenergic signaling can enhance effector T-cell activity and improve tumor control and immunotherapy responses in these models, reflecting a role for neural-immune modulation of tumor immunity [96,97].

Future investigations could determine the relative contributions of distinct neuronal subtypes—sensory, sympathetic, or parasympathetic—to tumor progression across different cancer types. It would also be important to define the temporal windows during which neural modulation exerts maximal impact and to assess the durability of tumor control following circuit-level interventions. If these parameters were clarified, neuromodulatory strategies might be integrated with systemic therapies in a rational and tumor-specific manner. In this framework, neuronal activity could be viewed not as an immutable feature of the tumor microenvironment, but as a biologically tunable variable whose precise adjustment might influence malignant trajectory.

### 5.4. Combined Therapies

The greatest clinical potential of neural-targeted strategies likely resides in rational combination regimens. Because neural signaling operates upstream of multiple oncogenic programs, its disruption may sensitize tumors to diverse therapeutic modalities while limiting adaptive escape [109]. Preclinical studies indicate that modulation of neural signaling can enhance the efficacy of established anticancer therapies. In preclinical tumor models, a reduction in β-adrenergic signaling increases effector CD8^+^ T-cell infiltration and can decrease regulatory T-cell accumulation within the tumor microenvironment, altering immune balance toward antitumor activity. This modulation of immunity by β-adrenergic blockade suggests potential synergy with immunotherapies such as anti-PD-1 agents, although combined therapy effects with measured T-cell infiltration and Treg reduction have not yet been published [97,131]. In small-cell lung cancer xenograft models, inhibition of NMDA-type glutamate receptors reduces tumor cell proliferation and increases apoptosis. When combined with chemotherapeutic agents such as topotecan, NMDA receptor blockade produces additive or synergistic antitumor effects relative to chemotherapy alone, indicating that targeting glutamatergic signaling can enhance the efficacy of conventional cytotoxic therapy [132]. Future studies will be required to determine whether the magnitude of therapeutic synergy depends on tumor type, neural subtype involvement, or treatment timing. It will also be valuable to assess whether neural modulation can prevent or delay the emergence of resistance under prolonged therapeutic pressure. If validated in clinical settings, combination strategies integrating neural-targeted interventions with systemic therapies could represent a structured means of enhancing treatment responsiveness.

### 5.5. Imaging the Neural–Tumor Axis: PET-Based Biomarkers

Translating the neural–tumor axis into clinically actionable metrics requires non-invasive tools capable of quantifying tumor-associated neural features in vivo. Positron emission tomography (PET) offers a molecular imaging platform with the potential to visualize biological processes relevant to neurotransmission and neuro–immune activation within tumors. Several PET tracer classes could, in principle, provide quantitative readouts of tumor-associated neural components. Radioligands targeting cholinergic terminals (for example, vesicular acetylcholine transporter [VAChT] imaging) and tracers of sympathetic nerve terminals (such as 11C-meta-hydroxyephedrine) are well established in non-oncologic settings and may be repurposed to probe neural signatures in solid tumors [133]. Although clinical PET studies directly assessing tumor innervation remain limited, these findings support the feasibility of imaging neural components within solid tumors.

In parallel, PET imaging of translocator protein (TSPO) has been applied in gliomas to capture neuroinflammatory activation, offering an indirect window into neural–immune crosstalk and tumor-associated microenvironment remodeling [134]. Importantly, it should be noted that tumor innervation imaging using PET remains at an early stage of development. Furthermore, tracer uptake may not exclusively reflect neuronal components but can also arise from immune and stromal cells within the tumor microenvironment, particularly in the context of neuroinflammation.

Although direct clinical quantification of tumor innervation remains limited, these imaging strategies suggest a feasible path toward neural biomarker development. PET-based metrics could support patient stratification according to neural dependency, monitor pharmacodynamic responses to neural-targeted therapies, and help identify tumors most likely to benefit from circuit-level interventions.

## 6. Conclusions

Cancer neuroscience reframes tumor biology by positioning the nervous system as a systems-level regulator of malignant progression. Rather than passive bystanders, neurons influence proliferative signaling, survival programs, angiogenesis, invasion, metabolic plasticity, and immune evasion. Tumor innervation therefore represents an important dimension of the malignant ecosystem, integrating electrical activity with transcriptional, metabolic, and immunological processes.

Therapeutically, targeting the neural–tumor axis extends beyond inhibition of individual pathways. Structural disruption of pseudo-synapses, pharmacological blockade of neurotransmission, circuit-level neuromodulation, interference with metabolic coupling, and rational combinatorial regimens collectively demonstrate that neural inputs are actionable determinants of tumor behavior in preclinical models. Progress toward clinical translation will require integration of mechanistic insight with quantitative biomarkers, refined neuromodulatory strategies, and identification of tumor contexts most dependent on neural circuitry. However, many aspects of tumor–neural communication remain incompletely understood, including the extent to which different neuronal subtypes contribute to tumor progression across cancer types.

Ultimately, recognition of the neural–tumor interface as an integrative dimension of cancer biology expands both conceptual and therapeutic frameworks. Continued rigorous investigation will determine the extent to which targeting neural regulation can be incorporated into precision oncology strategies.

## Figures and Tables

**Table 1:** Neural regulation across the hallmarks of cancer and associated signaling pathways. Summary of key neural inputs, signaling pathways, and representative mechanisms.

Hallmark of Cancer	Neural Input	Key Signaling Pathways	Representative Mechanisms
Sustaining proliferative signaling	Glutamatergic, cholinergic, adrenergic signaling	MAPK/ERK; PI3K/AKT; CaMKII; cAMP/PKA	Calcium influx, CREB activation, cyclin upregulation
Evading growth suppressors and resisting cell death	Adrenergic, cholinergic, glutamatergic signaling	PI3K/AKT; ERK; NF-κB; p53 modulation	BCL-2 family regulation, p53 inhibition, anti-apoptotic signaling
Inducing angiogenesis	Adrenergic, cholinergic, sensory neuropeptides	cAMP/PKA; HIF1α; VEGF signaling	VEGF induction, endothelial activation, NO production
Enabling invasion and metastasis	Glutamatergic and cholinergic inputs	Ca^2+^ signaling; Rho GTPases; MMP activation	Cytoskeletal remodeling, invadopodia formation, ECM degradation
Reprogramming tumor metabolism	Glutamatergic, adrenergic signaling	CaMKII; AMPK; mTOR; OXPHOS regulation	Mitochondrial transfer, metabolic coupling, lipid mobilization
Modulating immune evasion	Adrenergic and sensory signaling	cAMP/PKA; STAT3; immune checkpoint pathways	PD-L1 regulation, Treg recruitment, macrophage polarization
Tumor-promoting inflammation and microenvironment remodeling	Neural–stromal interactions	Cytokine signaling; NF-κB; IL-6 pathways	Fibroblast activation, Schwann cell recruitment, ECM remodeling

## Data Availability

No new data were created or analyzed in this study.
